# Supplementary material for: Fungal Endophytes: Discovering What Lies within Some of Canada’s Oldest and Most Resilient Grapevines
Source: J Fungi (Basel). 2024 Jan 26;10(2):105. doi: 10.3390/jof10020105 (PMC10890244; doi:10.3390/jof10020105)
Supplement: Supplementary file 1 [file jof-10-00105-s001.zip › Supplementary Figure S1-S7.pptx]

## Slide 1
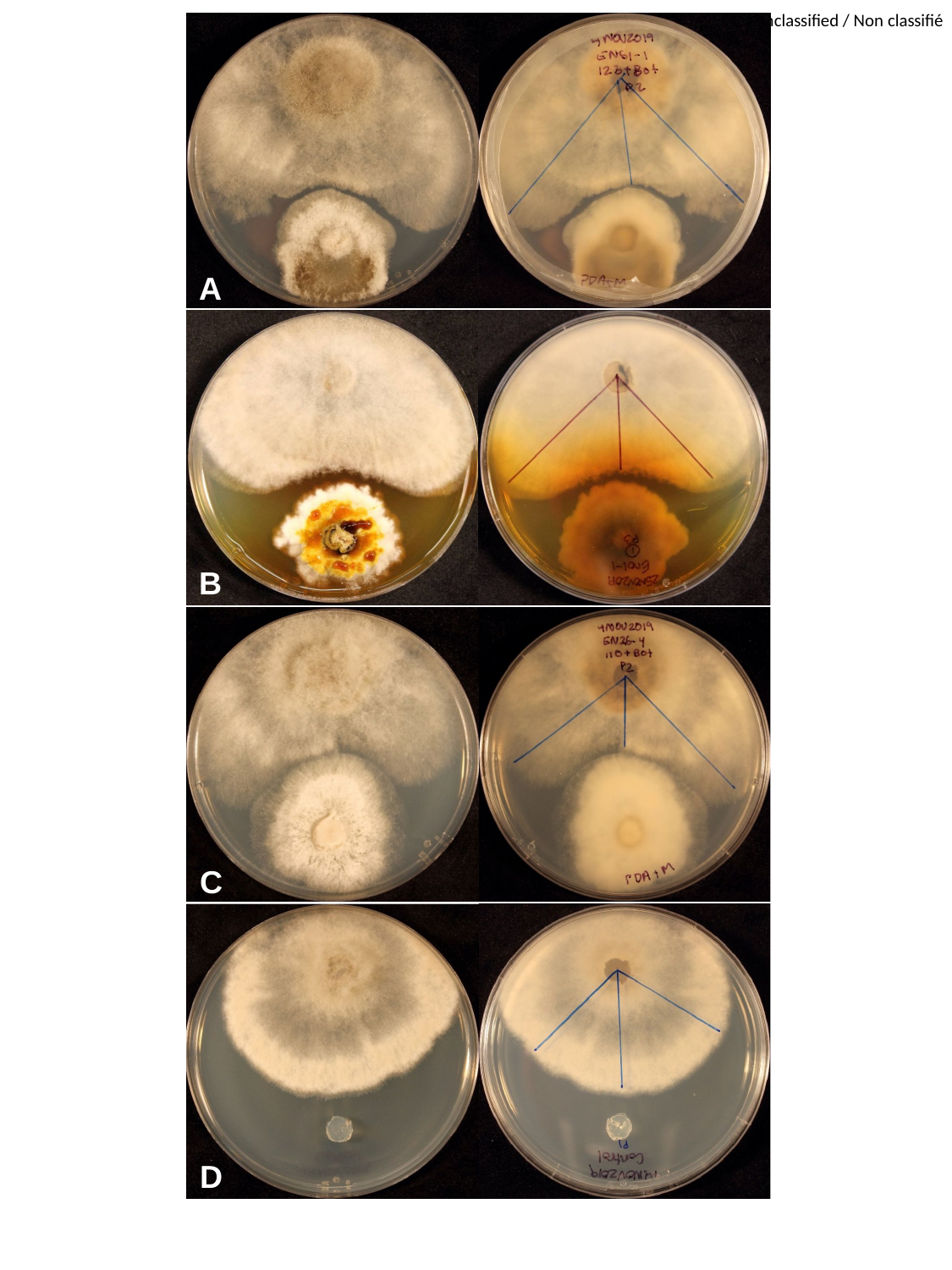

A
B
C
D

## Slide 2
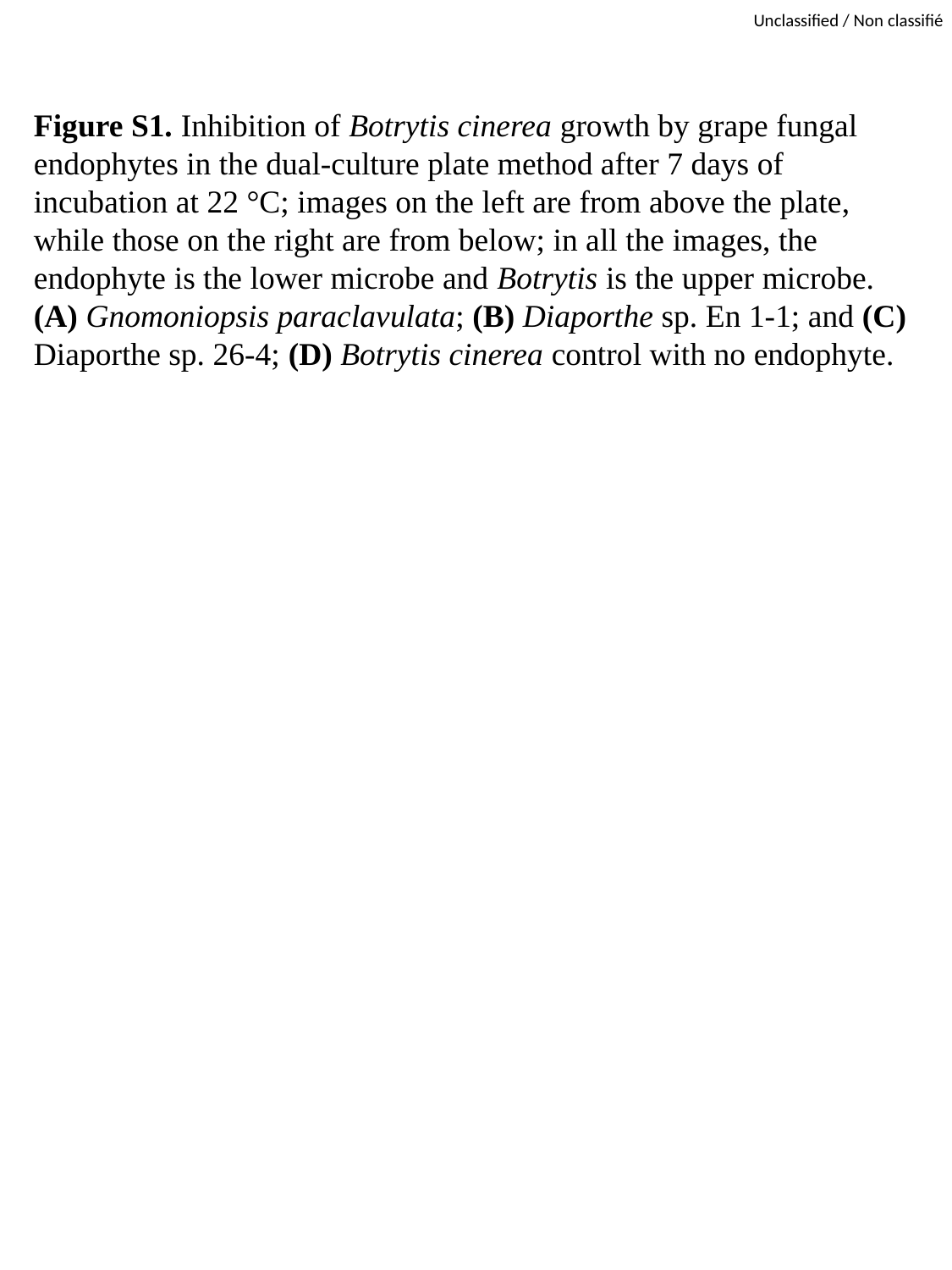

Figure S1. Inhibition of Botrytis cinerea growth by grape fungal endophytes in the dual-culture plate method after 7 days of incubation at 22 °C; images on the left are from above the plate, while those on the right are from below; in all the images, the endophyte is the lower microbe and Botrytis is the upper microbe. (A) Gnomoniopsis paraclavulata; (B) Diaporthe sp. En 1-1; and (C) Diaporthe sp. 26-4; (D) Botrytis cinerea control with no endophyte.

## Slide 3
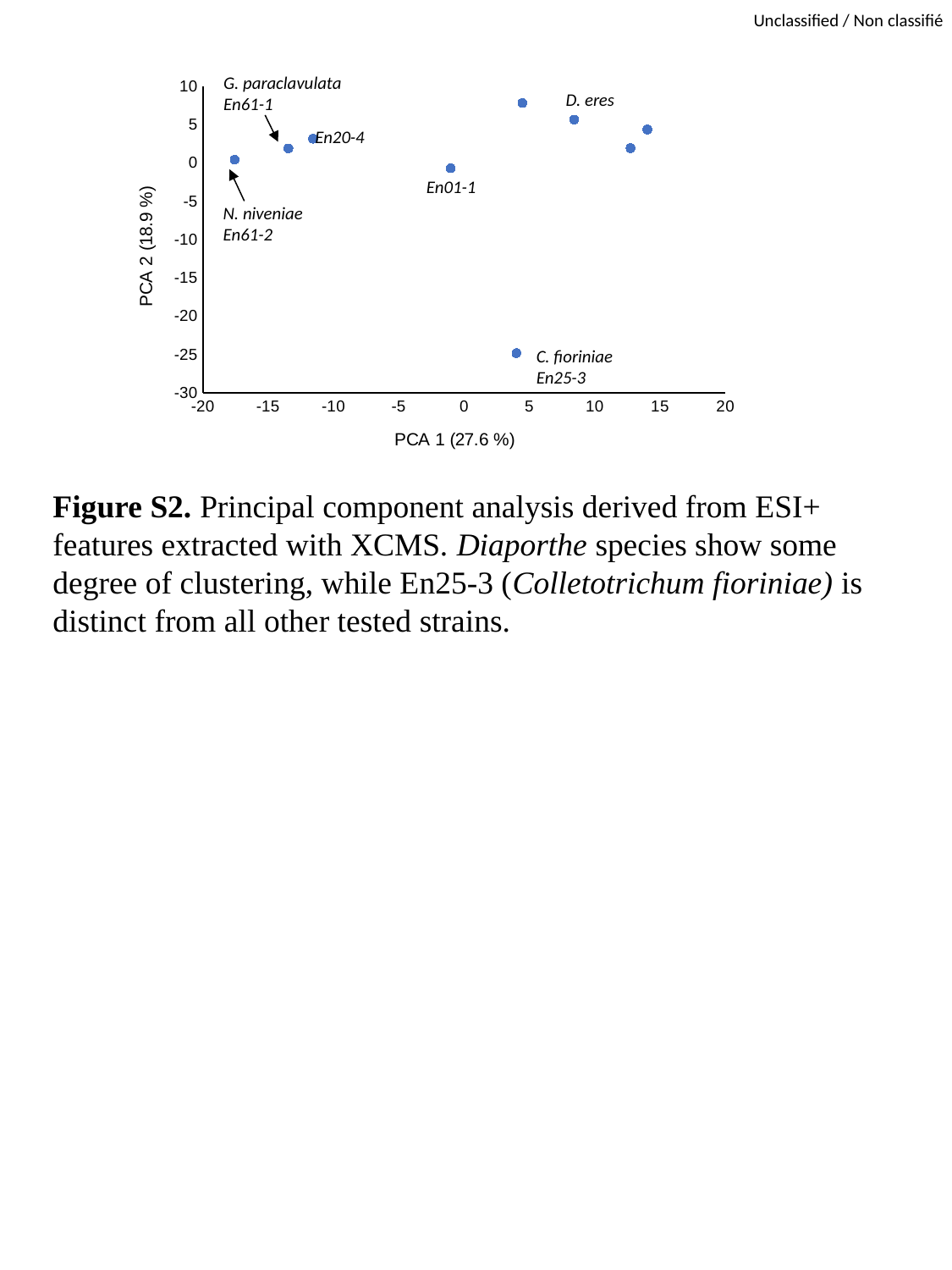

G. paraclavulata
En61-1
### Chart
| Category | C.fioriniae |
|---|---|D. eres
En20-4
En01-1
N. niveniae
En61-2
C. fioriniae
En25-3
Figure S2. Principal component analysis derived from ESI+ features extracted with XCMS. Diaporthe species show some degree of clustering, while En25-3 (Colletotrichum fioriniae) is distinct from all other tested strains.

## Slide 4
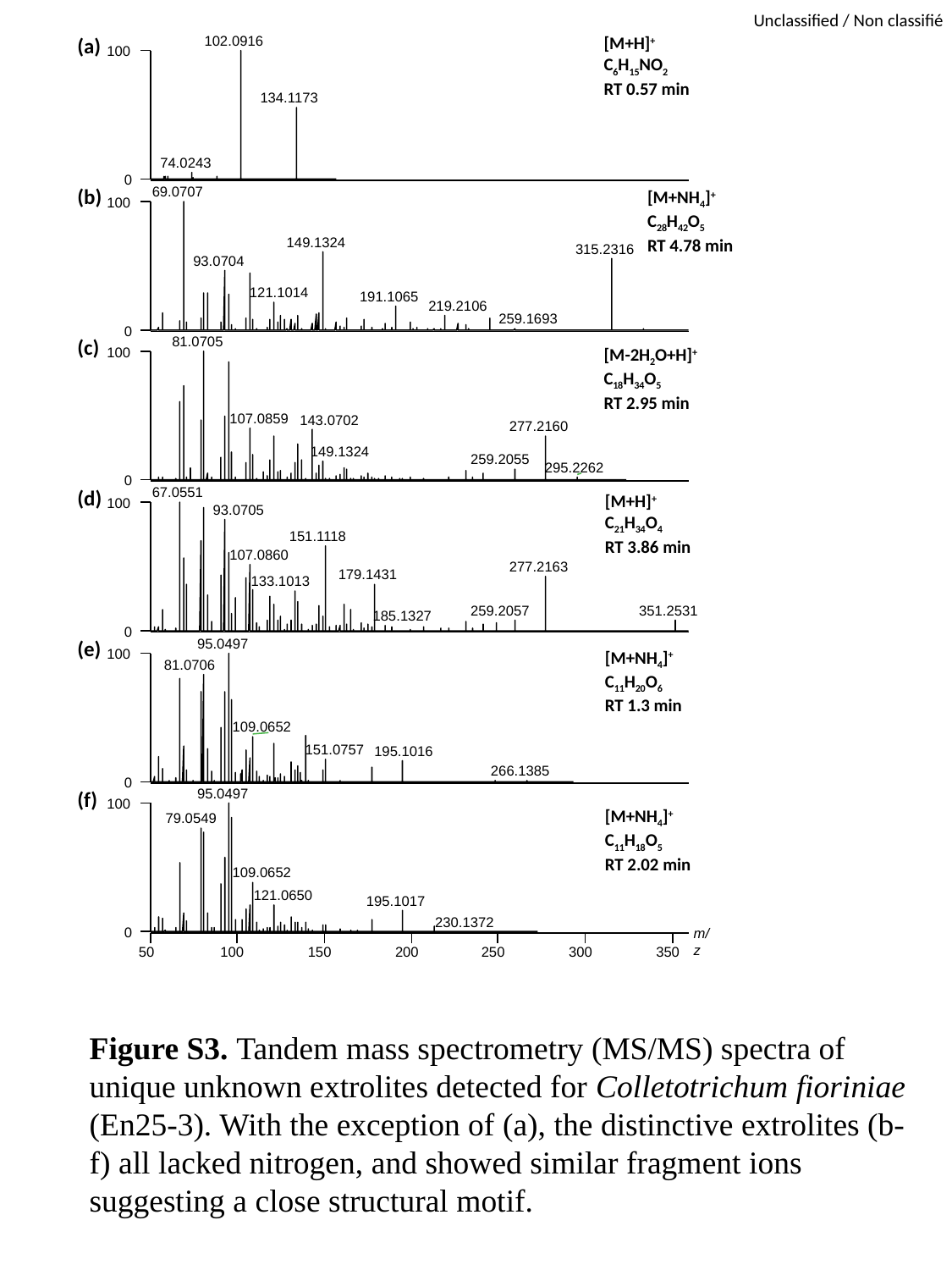

(a)
[M+H]+
C6H15NO2
RT 0.57 min
102.0916
100
134.1173
74.0243
0
(b)
[M+NH4]+
C28H42O5
RT 4.78 min
69.0707
100
149.1324
315.2316
93.0704
121.1014
191.1065
219.2106
259.1693
0
(c)
81.0705
[M-2H2O+H]+
C18H34O5
RT 2.95 min
100
107.0859
143.0702
277.2160
149.1324
259.2055
295.2262
0
(d)
[M+H]+
C21H34O4
RT 3.86 min
67.0551
100
93.0705
151.1118
107.0860
277.2163
179.1431
133.1013
259.2057
351.2531
185.1327
0
(e)
95.0497
[M+NH4]+
C11H20O6
RT 1.3 min
100
81.0706
109.0652
151.0757
195.1016
266.1385
0
(f)
95.0497
100
[M+NH4]+
C11H18O5
RT 2.02 min
79.0549
109.0652
121.0650
195.1017
230.1372
0
m/z
50
100
150
200
250
300
350
Figure S3. Tandem mass spectrometry (MS/MS) spectra of unique unknown extrolites detected for Colletotrichum fioriniae (En25-3). With the exception of (a), the distinctive extrolites (b-f) all lacked nitrogen, and showed similar fragment ions suggesting a close structural motif.

## Slide 5
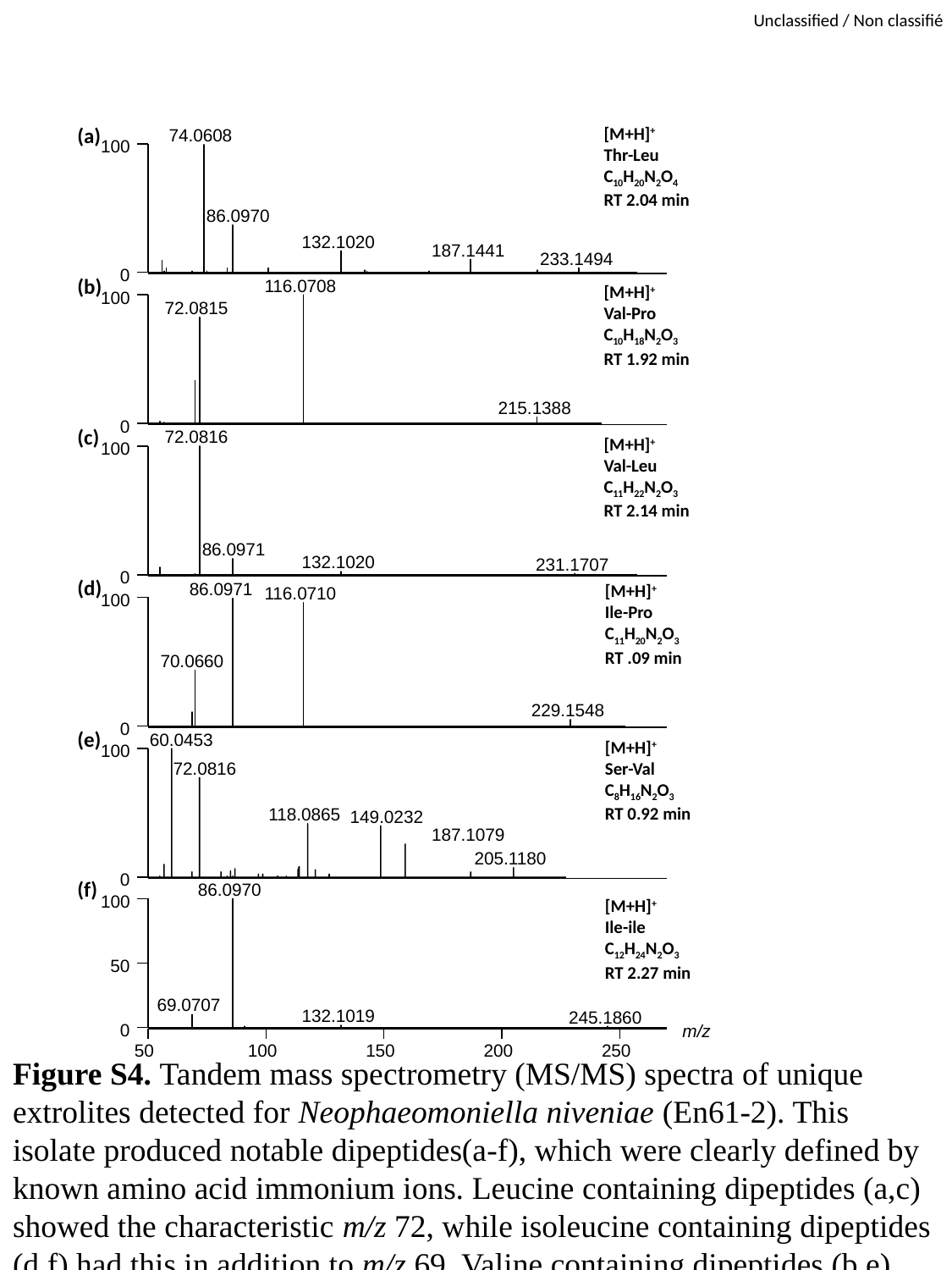

(a)
[M+H]+
Thr-Leu
C10H20N2O4
RT 2.04 min
74.0608
100
86.0970
132.1020
187.1441
233.1494
0
(b)
116.0708
[M+H]+
Val-Pro
C10H18N2O3
RT 1.92 min
100
72.0815
215.1388
0
(c)
72.0816
[M+H]+
Val-Leu
C11H22N2O3
RT 2.14 min
100
86.0971
132.1020
231.1707
0
(d)
[M+H]+
Ile-Pro
C11H20N2O3
RT .09 min
86.0971
116.0710
100
70.0660
229.1548
0
(e)
60.0453
[M+H]+
Ser-Val
C8H16N2O3
RT 0.92 min
100
72.0816
118.0865
149.0232
187.1079
205.1180
0
(f)
86.0970
[M+H]+
Ile-ile
C12H24N2O3
RT 2.27 min
100
50
69.0707
132.1019
245.1860
0
m/z
50
100
150
200
250
Figure S4. Tandem mass spectrometry (MS/MS) spectra of unique extrolites detected for Neophaeomoniella niveniae (En61-2). This isolate produced notable dipeptides(a-f), which were clearly defined by known amino acid immonium ions. Leucine containing dipeptides (a,c) showed the characteristic m/z 72, while isoleucine containing dipeptides (d,f) had this in addition to m/z 69. Valine containing dipeptides (b,e) showed m/z 72

## Slide 6
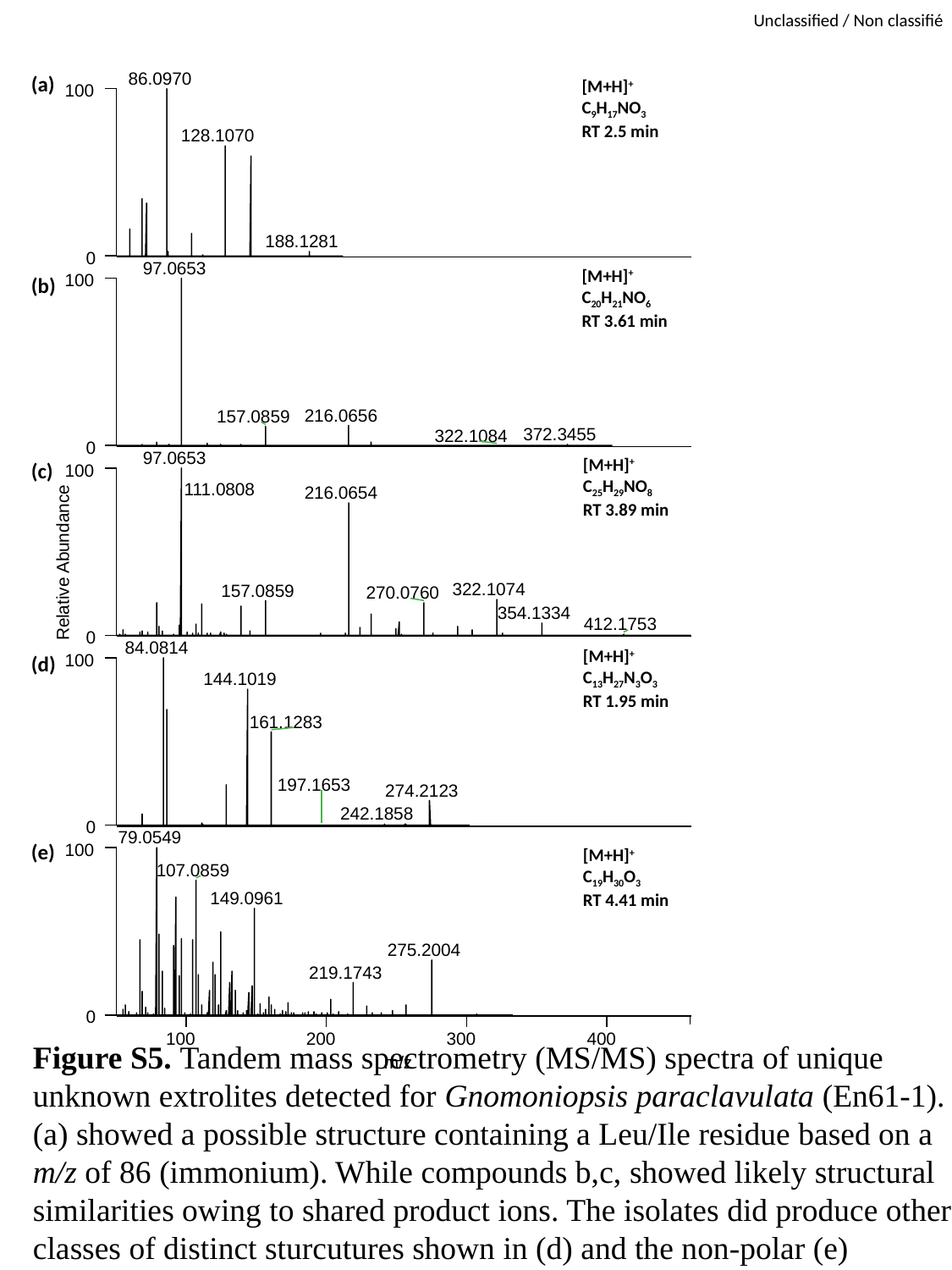

(a)
86.0970
[M+H]+
C9H17NO3
RT 2.5 min
100
128.1070
188.1281
0
97.0653
[M+H]+
C20H21NO6
RT 3.61 min
(b)
100
216.0656
157.0859
372.3455
322.1084
0
[M+H]+
C25H29NO8
RT 3.89 min
97.0653
(c)
100
111.0808
216.0654
Relative Abundance
322.1074
157.0859
270.0760
354.1334
412.1753
0
84.0814
[M+H]+
C13H27N3O3
RT 1.95 min
(d)
100
144.1019
161.1283
197.1653
274.2123
242.1858
0
79.0549
(e)
[M+H]+
C19H30O3
RT 4.41 min
100
107.0859
149.0961
275.2004
219.1743
0
100
200
300
400
Figure S5. Tandem mass spectrometry (MS/MS) spectra of unique unknown extrolites detected for Gnomoniopsis paraclavulata (En61-1). (a) showed a possible structure containing a Leu/Ile residue based on a m/z of 86 (immonium). While compounds b,c, showed likely structural similarities owing to shared product ions. The isolates did produce other classes of distinct sturcutures shown in (d) and the non-polar (e)
m/z

## Slide 7
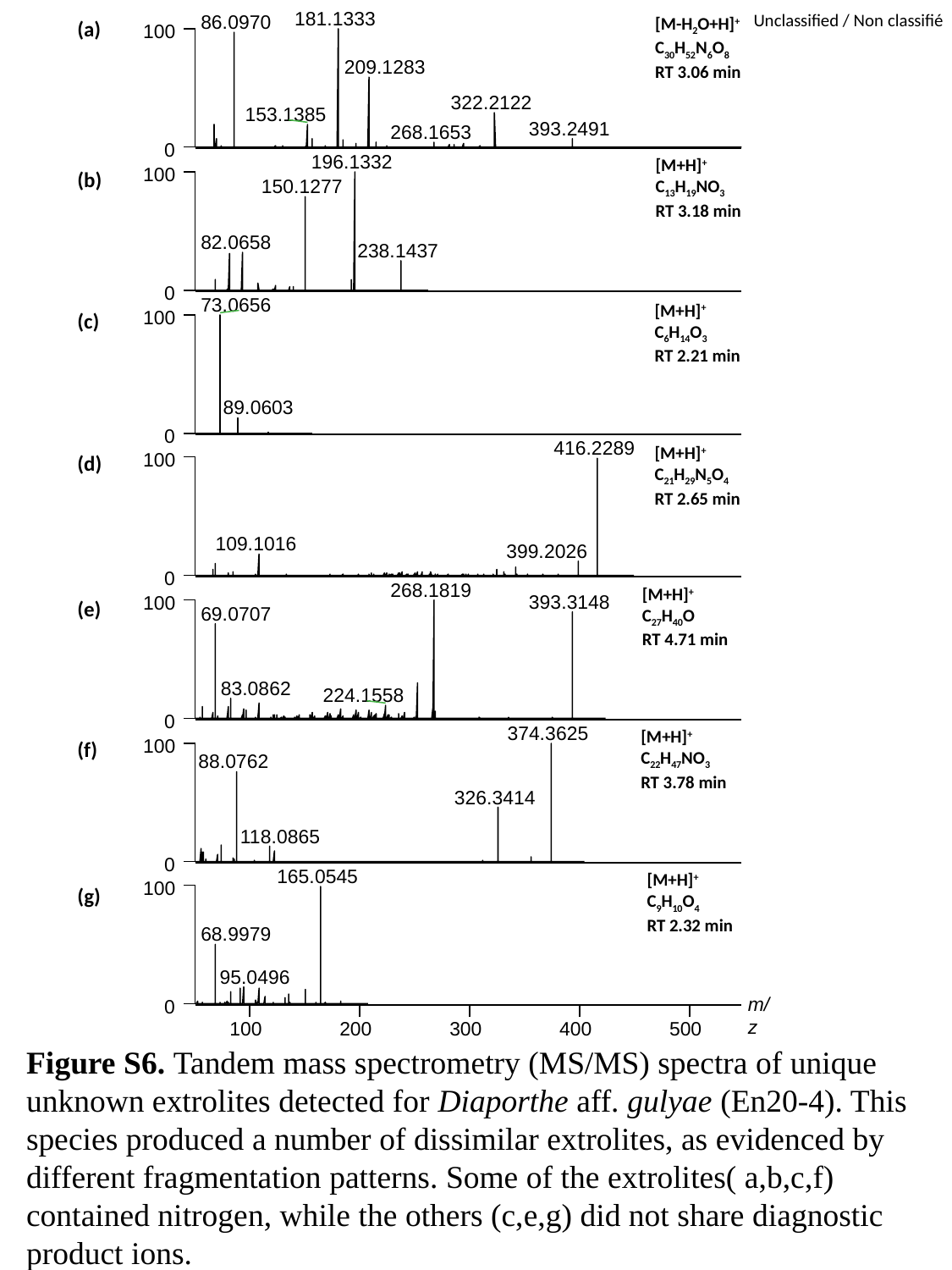

[M-H2O+H]+
C30H52N6O8
RT 3.06 min
181.1333
(a)
86.0970
100
209.1283
322.2122
153.1385
393.2491
268.1653
0
[M+H]+
C13H19NO3
RT 3.18 min
196.1332
(b)
100
150.1277
82.0658
238.1437
0
73.0656
[M+H]+
C6H14O3
RT 2.21 min
(c)
100
89.0603
0
[M+H]+
C21H29N5O4
RT 2.65 min
416.2289
(d)
100
109.1016
399.2026
0
[M+H]+
C27H40O
RT 4.71 min
268.1819
(e)
393.3148
100
69.0707
83.0862
224.1558
0
[M+H]+
C22H47NO3
RT 3.78 min
374.3625
(f)
100
88.0762
326.3414
118.0865
0
[M+H]+
C9H10O4
RT 2.32 min
165.0545
(g)
100
68.9979
95.0496
m/z
0
100
200
300
400
500
Figure S6. Tandem mass spectrometry (MS/MS) spectra of unique unknown extrolites detected for Diaporthe aff. gulyae (En20-4). This species produced a number of dissimilar extrolites, as evidenced by different fragmentation patterns. Some of the extrolites( a,b,c,f) contained nitrogen, while the others (c,e,g) did not share diagnostic product ions.

## Slide 8
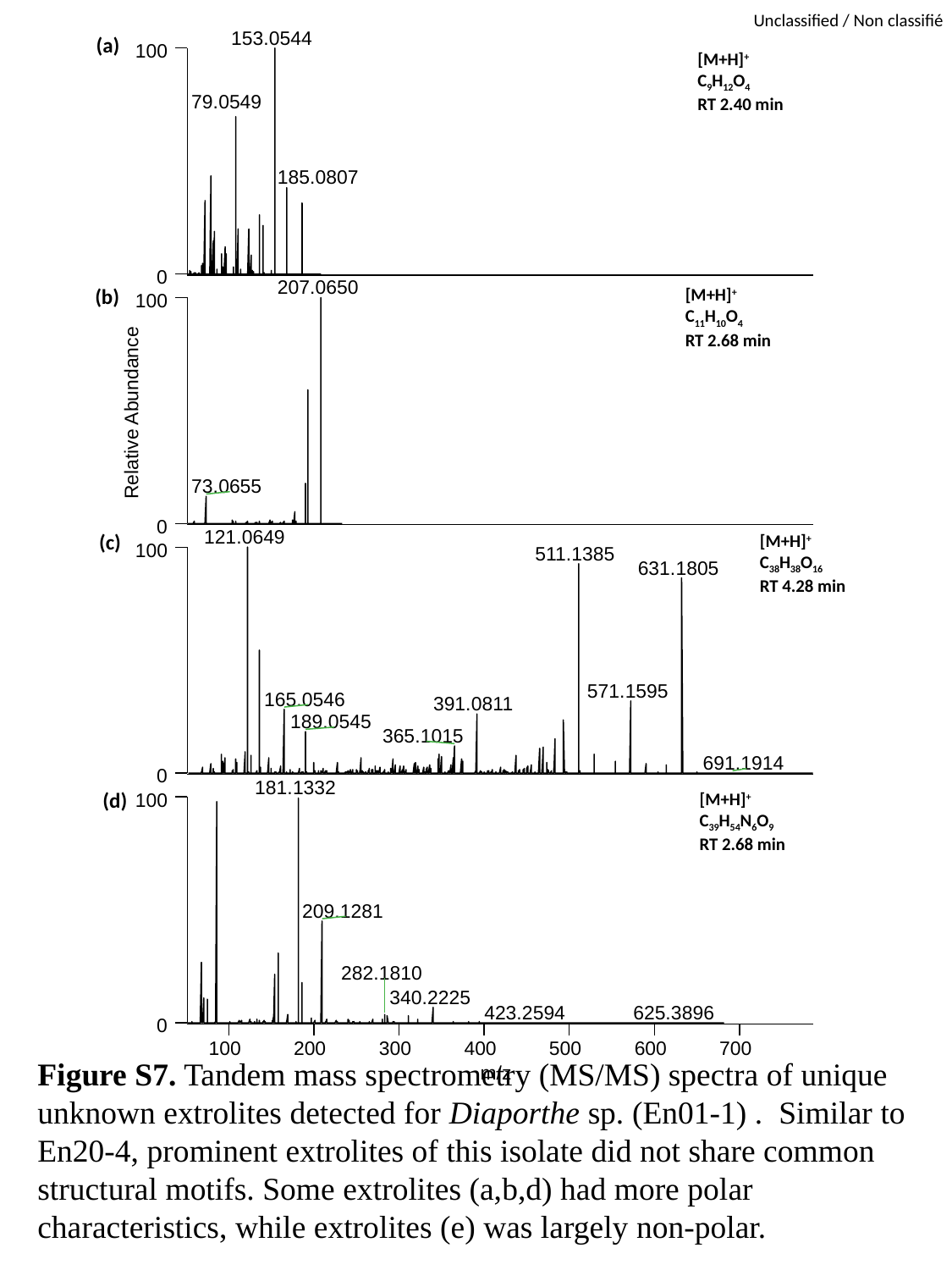

(a)
153.0544
100
[M+H]+
C9H12O4
RT 2.40 min
79.0549
185.0807
0
207.0650
(b)
[M+H]+
C11H10O4
RT 2.68 min
100
Relative Abundance
73.0655
0
(c)
[M+H]+
C38H38O16
RT 4.28 min
121.0649
100
511.1385
631.1805
571.1595
165.0546
391.0811
189.0545
365.1015
691.1914
0
181.1332
(d)
[M+H]+
C39H54N6O9
RT 2.68 min
100
209.1281
282.1810
340.2225
423.2594
625.3896
0
100
200
300
400
500
600
700
Figure S7. Tandem mass spectrometry (MS/MS) spectra of unique unknown extrolites detected for Diaporthe sp. (En01-1) . Similar to En20-4, prominent extrolites of this isolate did not share common structural motifs. Some extrolites (a,b,d) had more polar characteristics, while extrolites (e) was largely non-polar.
m/z
