# Supplementary material for: Fungal Endophytes: Discovering What Lies within Some of Canada’s Oldest and Most Resilient Grapevines
Source: J Fungi (Basel). 2024 Jan 26;10(2):105. doi: 10.3390/jof10020105 (PMC10890244; doi:10.3390/jof10020105)
Supplement: Supplementary file 1 [file jof-10-00105-s001.zip › Table S1 Sequences of forward and reverse primers of seven gene loci used in PCR amplification and sequencing for identification fungal endophytes.pdf]

TABLE S1. Sequences of forward and reverse primers of seven loci used in PCR amplification and sequencing for identification of fungal endophytes isolated from grapevine.

**Locus**	**Primer**	**Sequence (5′ to 3′)**	**Reference**
TUB2[a]	T1<br>β-Sandy-R	AACATGCGTGAGATTGTAAGT<br>GCRCGNGGVACRTACTTGTT	O'Donnell and Cigelnik (1997)<br>Stukenbrock et al. (2012)
ITS[b]	ITS1<br>ITS4	TCCGTAGGTGAACCTGCGG<br>TCCTCCGCTTATTGATATGC	White et al. (1990)<br>White et al. (1990)
TEF-1α[c]	EF1-low<br>EF1-low	GARGTACCAGTSATCATGTTCTT<br>GARGTACCAGTSATCATGTTCTT	O'Donnell et al. 1998<br>O'Donnell et al. 1998
RPB2[d]	fRPB2-5F<br>fRPB2-414R	GAYGAYMGWGATCAYTTYGG<br>ACMANNCCCCARTGNGWRTTRTG	Liu et al. (1999)<br>Quaedvlieg et al. (2011)
ACT[e]	ACT-512F<br>ACT2Rd	ATGTGCAAGGCCGGTTTCGC<br>ARRTCRCGDCCRGCCATGTC	Carbone & Kohn (1999)<br>Groenewald et al. (2012
CHS-1[f]	CHS-79F<br>CHS-79R	TGGGGCAAGGATGCTTGGAAGAAG<br>TGGAAGAACCATCTGTGAGAGTTG	Carbone and Kohn (1999)<br>Carbone and Kohn (1999)
LSU[g]	LSU1Fd<br>LR5	GRATCAGGTAGGRATACCCG<br>TCCTGAGGGAAACTTCG	Crous et al. (2009a)<br>Vilgalys & Hester (1990)

[a]β-tubulin, [b]Internal transcribed spacer (ITS) region of 5.8 S ribosomal DNA, [c]Part of the translation elongation factor 1 alpha, [d]RNA polymerase second largest subunit, [e]Partial actin, [f]Chitin synthase I and [g]28S nuclear ribosomal DNA gene.
