# Supplementary material for: Fungal Endophytes: Discovering What Lies within Some of Canada’s Oldest and Most Resilient Grapevines
Source: J Fungi (Basel). 2024 Jan 26;10(2):105. doi: 10.3390/jof10020105 (PMC10890244; doi:10.3390/jof10020105)
Supplement: Supplementary file 1 [file jof-10-00105-s001.zip › Table S2. Extrolite screening of bioactive compounds produced fungal endophytes strains.pdf]

**Compound**	**Formula**	***D. eres*** (# of strains)	***D. sp.* - En01.1**	**D. aff. *gulyae* En20.4**	***C. fioriniae* En25.3**	***G. paraclavulata* En61.1**	***N. niveniae* En61.2**
proline betaine	$C_7H_{13}NO_2$	+ (4)	+	-	-	-	-
tryptamine	$C_{10}H_{12}N_2$	+ (3)	-	-	-	-	-
hypoxyanthine	$C_5H_4N_4O$	+ (4)	+	-	-	-	-
phenylethylamine	$C_8H_{11}N$	+ (2)	-	-	-	-	-
choline sulfate	$C_5H_{13}NO_4S$	+ (2)	-	-	+	+	+
phosphocholine	$C_{31}H_{54}NO_6P$	+ (1)	-	-	-	-	-
-	$C_{18}H_{33}NO_2$	+ (4)	-	-	-	-	-
-	$C_{18}H_{35}NO_2$	+ (4)	-	-	-	-	-
-	$C_{18}H_{33}NO$	+ (3)	-	-	-	-	-
-	$C_{18}H_{35}NO$	+ (3)	-	-	-	-	-
-	$C_{12}H_{17}NO$	+ (3)	-	-	-	-	-
-	$C_{12}H_{14}N_2O$	+ (3)	-	-	-	-	-
Nectriapyrone	$C_{11}H_{14}O_3$	+ (4)	-	+	-	-	-
-	$C_{10}H_{13}NO$	+ (3)	-	-	-	-	-
-	$C_{18}H_{28}O_3$	+ (2)	+	-	+	-	-
-	$C_9H_{12}O_4$	+ (2)	+	-	-	-	-
-	$C_{13}H_{27}N_3O_3$	+ (2)	-	-	-	+	-
-	$C_6H_{15}NO_2$	+ (1)	-	-	+	-	-
-	$C_5H_{13}NO$	+ (1)	-	-	-	-	+
-	$C_{31}H_{56}NO_6P$	+ (1)	-	-	-	-	-
-	$C_{10}H_{10}O_3$	+ (1)	-	-	-	-	-
-	$C_{12}H_{14}O_3$	+ (1)	-	-	-	-	-
Desmethylnectriapyrone	$C_{10}H_{12}O_3$	+ (1)	-	-	-	-	-
-	$C_{15}H_{20}O_5$	+ (1)	-	-	-	-	-
-	$C_{15}H_{20}O_6$	+ (1)	-	-	-	-	-
-	$C_{20}H_{43}NO_2$	+ (1)	-	-	-	+	+
-	$C_{30}H_{52}N_6O_8$	-	+	+	-	-	-
-	$C_{13}H_{19}NO_3$	-	+	+	-	-	-
-	$C_{27}H_{40}O$	-	-	+	-	-	+
-	$C_6H_{14}O_3$	-	-	+	-	-	-
-	$C_{21}H_{29}N_5O_4$	-	-	+	-	-	-
-	$C_{22}H_{47}NO_3$	-	-	+	-	-	-
-	$C_9H_{10}O_4$	-	-	+	-	-	-
-	$C_{38}H_{38}O_{16}$	-	+	-	-	-	-
-	$C_{11}H_{10}O_4$	-	+	-	-	-	-
-	$C_{39}H_{54}N_6O_9$	-	+	-	-	-	-
carnitine	$C_7H_{15}NO_3$	-	-	-	+	-	+
-	$C_{21}H_{34}O_4$	-	-	-	+	-	-
-	$C_{11}H_{20}O_6$	-	-	-	+	-	-
-	$C_{11}H_{18}O_5$	-	-	-	+	-	-
-	$C_{18}H_{34}O_5$	-	-	-	+	-	-
-	$C_{25}H_{29}NO_8$	-	-	-	-	+	+
-	$C_{20}H_{21}NO_6$	-	-	-	-	+	+
-	$C_9H_{17}NO_3$	-	-	-	-	+	+
-	$C_{19}H_{30}O_3$	-	-	-	-	+	-
Ile/Phe	$C_{15}H_{22}N_2O_3$	-	-	-	-	+	+

Ile/Ile	$C_{12}H_{24}N_2O_3$	-	-	-	-	-	+
Ser/Val	$C_8H_{16}N_2O_4$	-	-	-	-	-	+
Ile/Pro	$C_{11}H_{20}N_2O_3$	-	-	-	-	-	+
Leu/Val	$C_{11}H_{22}N_2O_3$	-	-	-	-	-	+
Leu/Thr	$C_{10}H_{20}N_2O_4$	-	-	-	-	-	+
-	$C_{18}H_{39}NO_3$	-	-	-	-	-	+

TABLE S2: Extrolite screening of bioactive compounds produced fungal endophytes strains
